# Advancements in pediatric obstructive sleep apnea: cognitive implications and the role of AI in precision medicine

**DOI:** 10.3389/fmed.2025.1704504

**Published:** 2025-11-21

**Authors:** Bomeng Zhao, Haixia Fan, Huiyan Niu, Yan Li, Shudan Deng, Lu Zhai, Limantian Wang, Xiaoling Gao

**Affiliations:** 1 The Second Hospital of Shanxi Medical University, Taiyuan, China; 2 First Hospital of Shanxi Medical University, Taiyuan, China; 3 Department of Sleep Center, The Second Hospital of Shanxi Medical University Taiyuan, Taiyuan, China

**Keywords:** pediatric obstructive sleep apnea, cognition, bibliometric analysis, artificial intelligence, precision medicine

## Abstract

**Background:**

Obstructive sleep apnea (OSA) in children is linked to cognitive impairment, which is further aggravated by fragmented sleep, intermittent hypoxia, and comorbid attention deficit hyperactivity disorder (ADHD), often resulting in poor academic performance, behavioral problems, and delayed neurodevelopment.

**Methods:**

A bibliometric and scientometric analysis was conducted using Web of Science Core Collection (WoSCC) and Scopus databases (1983–2025). After exclusion of non-article records and duplicates, 1,610 studies were included. CiteSpace, VOSviewer, and the R-based Bibliometrix package were employed to analyze publication trends, authorship, institutional collaborations, co-citations, and keyword evolution.

**Results:**

Since 2010, scholarly attention to pediatric obstructive sleep apnea and its cognitive implications has grown rapidly, with research output increasing at an annual rate of 11.96%. The United States, China, and several European countries stand out as major contributors, both in terms of publications and international collaboration. Prominent academic centers—such as Harvard University, the University of Michigan, and the University of Chicago—serve as key institutional hubs. Among the most influential contributors to the field are David Gozal and Leila Kheirandish-Gozal, while leading journals include *Sleep Medicine* and *the Journal of Clinical Sleep Medicine*. Keyword mapping highlights the central focus on “obstructive sleep apnea,” “children,” and “sleep-disordered breathing,” alongside diagnostic and treatment terms such as “polysomnography” and “adenotonsillectomy.” Notably, there is growing emphasis on cognitive and behavioral aspects, including “cognition,” “behavior,” and “ADHD,” as well as comorbidities like “obesity” and “Down syndrome.” Recent clusters underscore advances in artificial intelligence (AI) and machine learning (ML) based models using oximetry, electrocardiogram (ECG), and acoustic data, enabling early detection and supporting precision medicine approaches.

**Conclusion:**

The relationship between pediatric obstructive sleep apnea and neurocognitive development has gained significant attention, with recent research focusing on cognitive outcomes and emerging technologies. While conventional treatments remain important, their limited impact on cognitive recovery underscores the need for early diagnosis and personalized therapies. Advances in artificial intelligence and machine learning are improving diagnostic accuracy, enabling earlier interventions, and supporting neurocognitive function in affected children. These developments reflect a shift toward precision-based, tailored care rather than one-size-fits-all treatments.

## 1 Introduction

Pediatric obstructive sleep apnea (OSA) is a prevalent condition that can profoundly impact children’s cognitive development, especially in areas such as attention, memory, and executive function. Even with treatment, many children continue to struggle significantly, often facing challenges in school performance, social relationships, and overall developmental progress (1–4). Key issues, including decreased attention, impaired working memory, and weakened executive function, are closely tied to sleep disturbances and frequent episodes of low oxygen levels. These disruptions interfere with vital brain processes necessary for memory formation and higher-order cognitive functions (5, 6). Comorbidities, especially Attention-Deficit/Hyperactivity Disorder (ADHD), often intensify these neurocognitive difficulties. The fragmentation of sleep can worsen ADHD symptoms, creating a cycle of inattention and executive dysfunction. This interaction further complicates clinical management, making it more challenging to address both conditions effectively (7).

Adenotonsillectomy (AT) and continuous positive airway pressure (CPAP) often lead to better sleep and a reduction in OSA symptoms, yet their impact on cognitive recovery remains inconsistent. This inconsistency highlights the need for more refined diagnostic methods, as early identification plays a crucial role in preventing lasting neurocognitive harm. Although polysomnography (PSG) continues to serve as the gold standard, it remains costly, time-intensive, and not always easily accessible (7). Recent developments in machine learning (ML) and artificial intelligence (AI) are reshaping how pediatric OSA is evaluated. By drawing on clinical signals—such as pulse oximetry, electrocardiogram (ECG) data, and acoustic features—these technologies support quicker and more accessible screening. This earlier identification may help reduce the risk of cognitive complications by allowing timely intervention (5, 6). In addition to improving diagnostic accuracy, AI-based precision medicine contributes to more individualized treatment strategies, enabling clinicians to adjust CPAP or AT according to each patient’s specific needs. By targeting both the disruption of sleep and the underlying causes of the condition, this tailored approach holds promise for enhancing neurocognitive outcomes (8).

Pediatric OSA is associated with enduring cognitive risks that may not fully resolve with conventional therapy. Advances in AI-driven diagnostics and treatment planning are beginning to create opportunities for earlier recognition and more individualized care. By supporting timely detection and tailored interventions, these approaches have the potential to improve cognitive outcomes for affected children.

## 2 Methods

### 2.1 Database and search strategy

The WoSCC is recognized for its stringent journal selection process and reliable citation tracking, which effectively capture the impact and reach of scholarly work. Scopus, on the other hand, stands out with its extensive disciplinary coverage and advanced citation tools, making it ideal for supporting interdisciplinary research. By combining WoSCC and Scopus, researchers gain a more comprehensive and precise bibliometric analysis, providing richer insights into research trends and academic developments.

**Figure 1:**
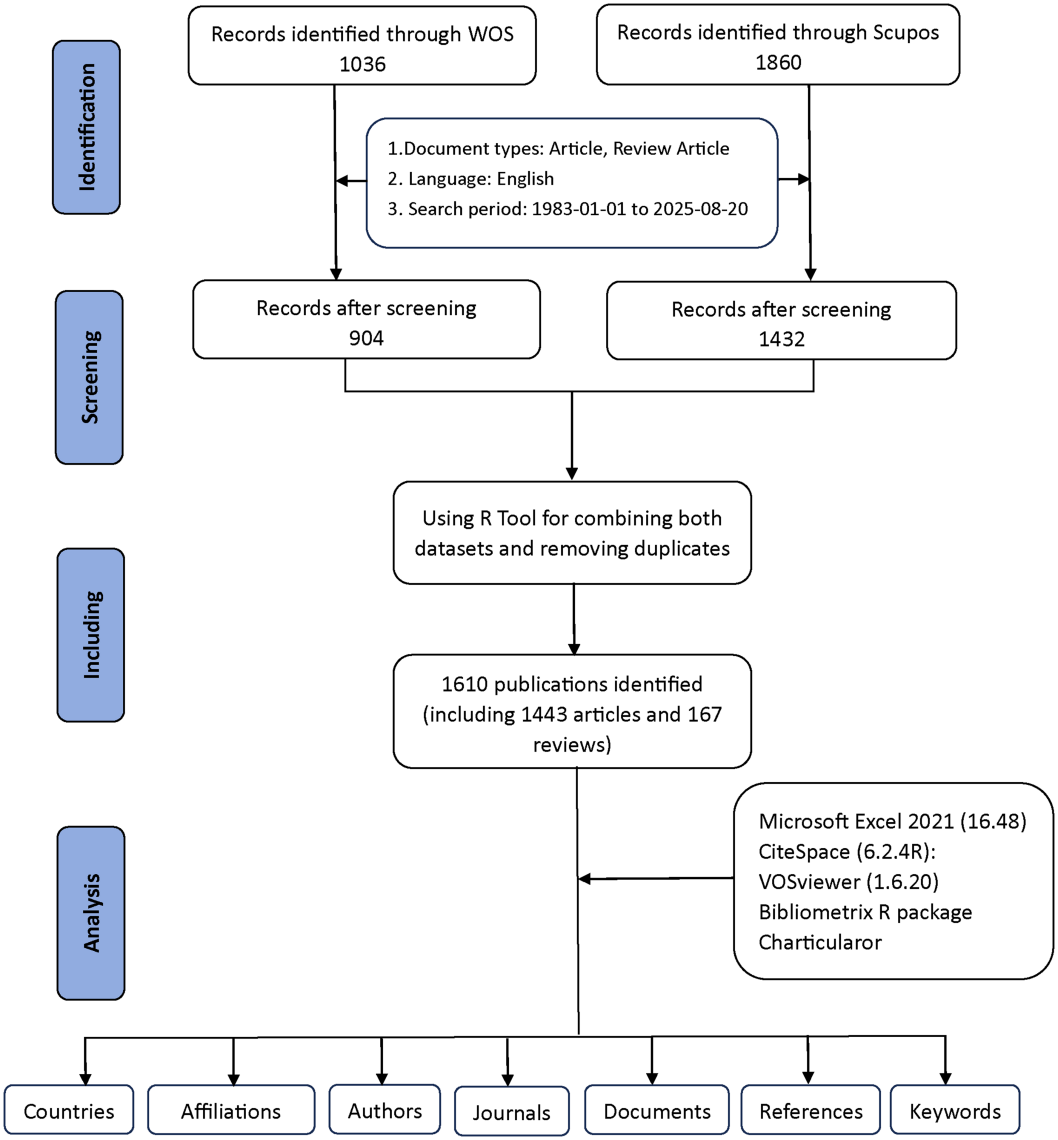
The flowchart for literature search, selection and analysis.

Figure 1 provides a detailed explanation of the data retrieval and exclusion criteria. Initially, to acquire bibliometric data, search terms were employed in the WoSCC and Scopus databases on August 20, 2025. An initial search was conducted using the title, abstract, and keywords of the publication. To choose relevant terms, we examined several prior publications in the literature (9, 10). Obstructive sleep apnea-related terms = (“OSAHS” OR “OSA “OR “OSAS” OR “obstructive sleep apnea hypopnea syndrome” OR “obstructive sleep apnea*“OR” Obstructive Sleep Apnea Syndrome” OR” Apnea*, Obstructive Sleep” OR “Sleep Apneas, Obstructive” OR “Sleep Apnea Hypopnea Syndrome” OR “Syndrome, Obstructive Sleep Apnea” OR “Syndrome, Sleep Apnea, Obstructive” OR “Sleep Apnea Syndrome, Obstructive” OR “Upper Airway Resistance Sleep Apnea Syndrome” OR “Syndrome, Upper Airway Resistance, Sleep Apnea” OR “snoring”). Child-related terms = (“child*” OR “pediatric*” OR “adolescen*” OR “infant*” OR “toddler*” OR “teen*” OR “youth”). Cognition-related terms = (“cognition” OR “cognitive function*” OR “neurocognit*” OR “executive function*” OR “attention” OR “memory” OR “learning” OR “IQ” OR “intelligence” OR “neuropsycholog* test*” OR “cognitive impair*” OR “cognitive deficit*”). By combining relevant keywords using the Boolean operator “AND” across the three categories—OSA-related terms AND child-related terms AND cognition-related terms—we conducted a targeted search. This ensured that the retrieved documents were relevant to the intersection of all three aspects: obstructive sleep apnea, children, and cognition. A total of 1,036 documents were retrieved from WoSCC and 1,860 from Scopus using this search strategy. Exclusions were made to eliminate items such as Proceeding Papers, Corrections, Early Access articles, News Items, Book Chapters, Retractions, Reprints, Biographical Items, Book Reviews, Meeting Abstracts, Editorial Materials, and Letters. Only articles and reviews published in English between 1983 and 2025 were kept. Ultimately, 904 documents remained in WoSCC, while Scopus retained 1,432 documents.

The two datasets were eventually merged with the help of the bibliometrix R package. Duplicates were mainly identified by comparing titles, author names, publication years, and DOIs. Where DOIs were missing, matches were determined based on a combination of the title and the first author’s name. After this automated process, potential duplicates were carefully reviewed by hand to ensure accuracy. This step helped prevent counting the same publication more than once across WoSCC and Scopus. As a result of this quality check, 1,610 unique records were kept for further analysis, including 1,443 articles and 167 reviews (904 from WoSCC and 706 from Scopus) (Figure 1). These procedures aimed to reduce duplication bias while maintaining the reliability of the data.

### 2.2 Data analysis procedures

#### 2.2.1 CiteSpace

CiteSpace (6.2.4R, 64-bit Advanced Edition) was employed for the data analysis (11). The data collection period spanned from January 1983 to August 2025, with a slice of one year. The nodes analyzed included authors, institutions, and keywords. The threshold for author and institution nodes was set to the top 25 per slice, without pruning. For keyword nodes, a threshold of 25 was applied with pruning using pathfinder and merged network techniques (12). Visual analyses were conducted to create knowledge maps for researchers, institutions, and keywords. All records obtained from WoSCC were exported as “full records and cited references” in plain text format.

#### 2.2.2 VOSviewer

VOSviewer (version 1.6.20), created by CWTS at Leiden University, was used to process the data (13). Full counting was applied, with thresholds set based on analytical items to generate visual representations of collaborative networks.

#### 2.2.3 Bibliometrix

The bibliometrix R package (https://www.bibliometrix.org), developed by Dr. Massimo Aria and Corrado Cuccurullo, was utilized for historiographic analysis, monitoring trends in journals and authors, and calculating bibliometric metrics such as the g-index (14), h-index (15), number of citations (NC), and number of publications (NP) (16).

#### 2.2.4 Other tools

Microsoft Excel 2021 (Version 16.48) was employed to organize the initial dataset. The Online Analysis Platform of Literature Metrology (https://bibliometric.com/) provided an intuitive interface for conducting bibliometric analysis of citation data.

## 3 Results

### 3.1 Annual publications trends

**Figure 2:**
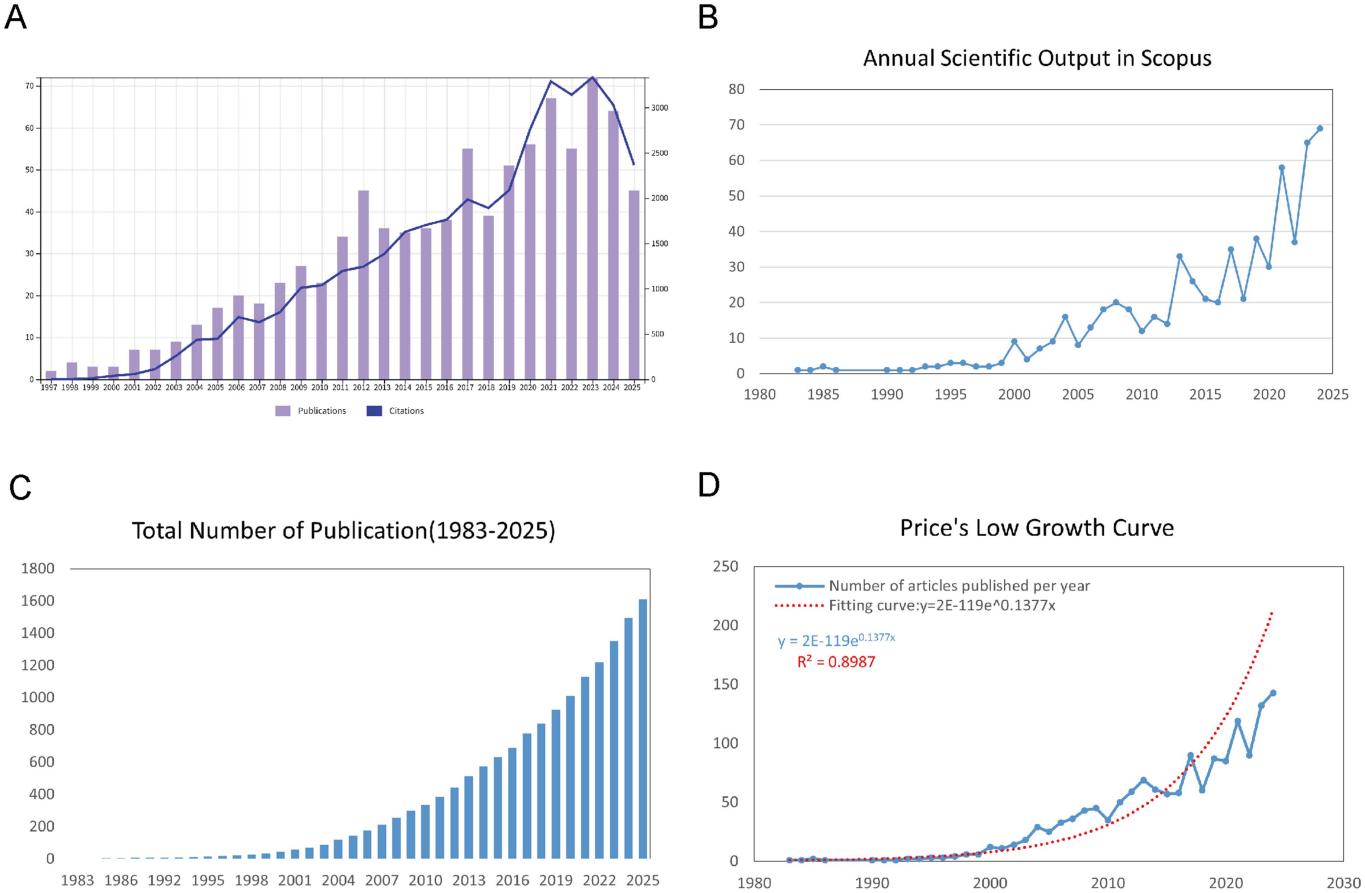
Overview of publications and citations on cognition in pediatric OSA (1983–2025). **(A)** Trends in annual publication numbers (bars) and citation counts (line) from the WoSCC database. **(B)** Yearly changes in publication numbers (bars) and citation figures (line) from the Scopus database. **(C)** Accumulated number of publications over time, illustrating the overall growth of the field. **(D)** Price’s Law curve fitting analysis of cumulative publications, demonstrating the exponential growth pattern of cognition in pediatric OSA research.

Our bibliometric analysis, spanning from 1983 to 2025, shows a significant rise in both the number of publications and citations within the field of childhood OSA and cognition. We retrieved 1,610 documents from WoSCC and Scopus, with an annual growth rate of 11.96%, reflecting a steady expansion of research in this area (Supplementary Table S1). As shown in Figure 2A, trends in the WoSCC reflect steady increases in both publications and citations from the 1990s onward, with a sharp upward trend emerging around 2015. Similarly, Figure 2B displays the annual trends in Scopus, confirming the upward trajectory of both publications and citations, especially after 2015. Figure 2C illustrates the cumulative growth of publications over time, showing a clear exponential increase, particularly in the last decade. To further quantify this growth, Figure 2D presents a Price’s Law growth curve fitting analysis, with the model equation y = 2E-119e^0.1377x^ and an R^2^ value of 0.8987, which demonstrates a strong alignment with the observed data. This analysis shows that the field has not only grown substantially in volume but also follows an exponential growth trend, typical of fast-evolving scientific areas.

### 3.2 Distributions of countries/regions

**Figure 3:**
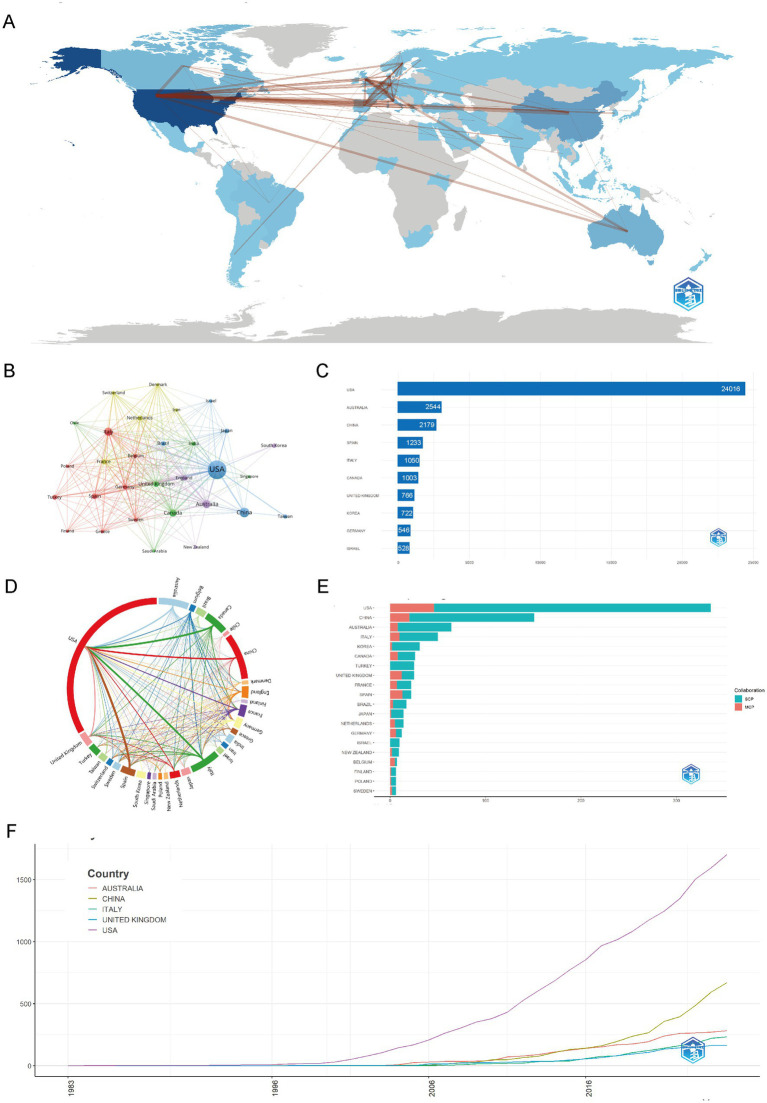
Global trends and collaboration networks in scientific publications on cognition in pediatric OSA (1983–2025). **(A)** Country/region collaboration map. The intensity of blue shading signifies higher collaboration rates, while the thickness of the connecting lines shows the strength of collaboration between countries. **(B)** Country clustering analysis (network). Nodes represent countries or regions, with their size proportional to the publication output. The thickness of the edges indicates the strength of co-authorship links between countries. Colors of the nodes show collaboration clusters found by the layout algorithm, where countries in the same color group have more frequent collaborations. Spatial closeness indicates stronger ties. **(C)** The top 10 countries/regions by citation impact are displayed. Horizontal bars illustrate the total citation numbers for publications linked to each country/region, with counts labeled on the bars. **(D)** Network map of national research output and cooperative relations (chord diagram). Every outer arc represents a country or region, with its length indicating the total collaborative output of that country within the network. Ribbons connect pairs of countries; ribbon width represents the strength of their collaboration (number of co-authored documents). Ribbon colors follow the color of the originating arc. **(E)** Leading countries by number of studies and collaboration type. Stacked horizontal bars display each country’s publication count, partitioned into single-country publications (SCP) and multiple-country publications (MCP). Teal segments denote SCP and salmon/red segments denote MCP (legend “Collaboration” in the panel). The MCP proportion indicates the extent of international collaboration. **(F)** Top 5 Countries/regions by publications. Lines show the cumulative number of publications from 1983 to 2025. The x-axis is year; the y-axis is cumulative document count.

As illustrated in Figures 3A,B,D, the analysis of the country collaboration network in childhood OSA and cognition research reveals a strong global network, with the USA at the center, closely collaborating with Europe, Australia, and Asia. These figures highlight key connections with Germany, France, China, and South Korea, demonstrating widespread international cooperation. Countries like Brazil, Canada, and Turkey are increasingly involved in both single-country and multi-country publications. This global collaboration underscores the field’s growing international engagement, facilitating knowledge exchange and advancing research on childhood OSA and cognition.

In Figures 3C,E,F, the distribution of countries and regions in childhood OSA and cognition research reveals a clear global leadership by the USA, which not only produces the highest number of publications but also leads in citations. China and Australia follow in terms of publication volume, with China contributing significantly to the growing body of research, particularly in recent years. Countries such as Italy, South Korea, and the United Kingdom also show substantial contributions, with increasing collaboration in multi-country publications. As shown in Supplementary Table S2 indicates that the USA leads in both single-country publications (SCP) and multiple-country publications (MCP), while countries like Spain, Brazil, and France show higher engagement in multi-country collaborations. Overall, the data highlights a growing global network, with research output heavily concentrated in North America, Europe, and Asia, demonstrating the worldwide focus on childhood OSA and cognition.

### 3.3 Distribution by institutions

**Figure 4:**
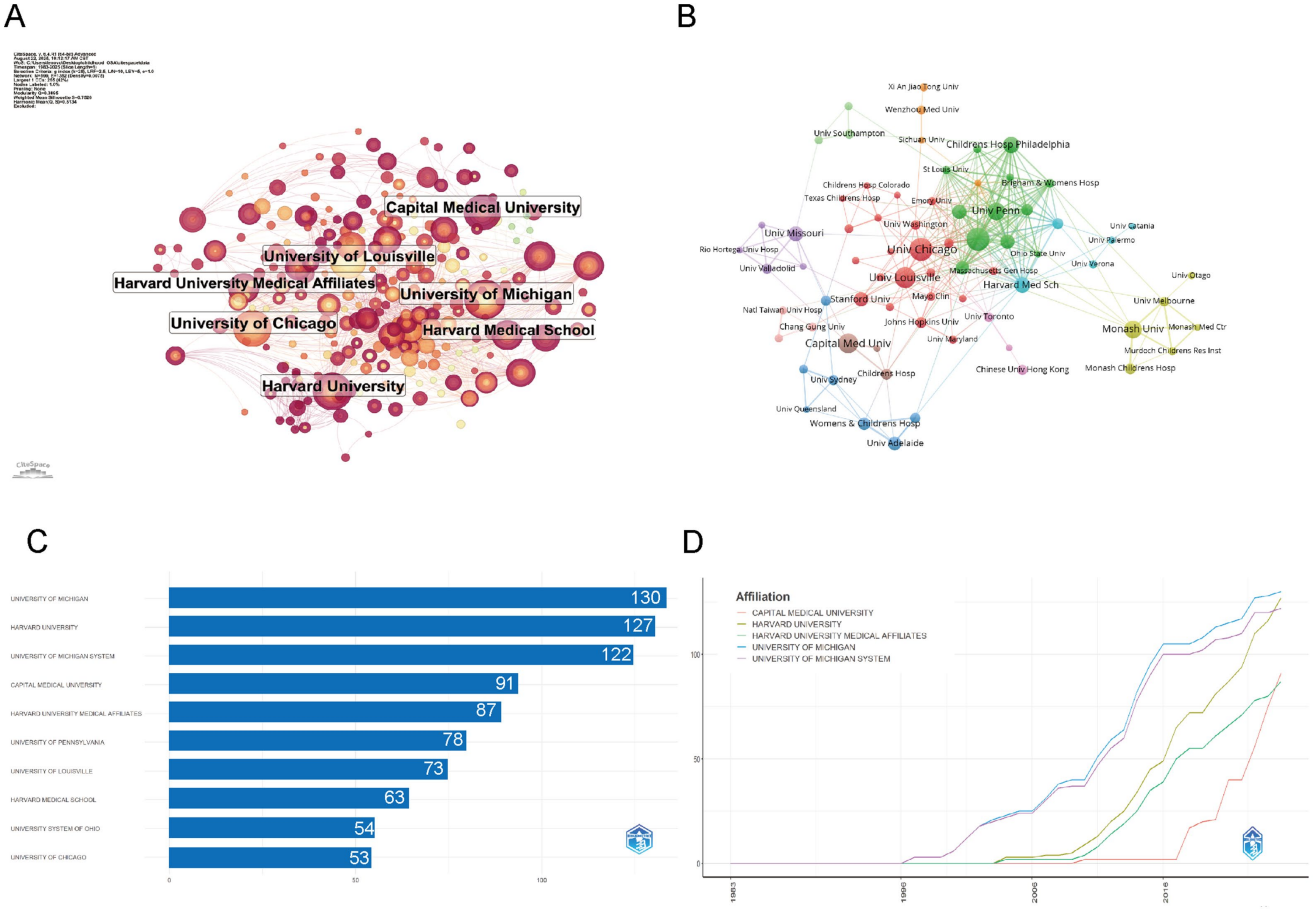
Institutional contributions and collaboration networks on cognition in pediatric OSA (1983–2025) **(A)** Research institution network. Nodes symbolize institutions, with their size showing the volume of publications and the thickness of lines representing the strength of collaborations. **(B)** Institutional collaboration network. Node size reflects the amount of collaboration activity, and unique colors signify clusters, often indicating regional or institutional affiliations. Line thickness reflects the strength of collaboration, as indicated by the count of shared publications. **(C)** Top 10 institutions by citation impact. **(D)** Institutional publication trends. The x-axis represents years, while the y-axis shows the number of publications.

The institutional collaboration network in childhood OSA and cognition research, depicted in Figure 4A, showcases key institutions such as Harvard University, University of Michigan, and University of Chicago. These institutions play central roles, as shown by their extensive network connections, underscoring their leadership and active collaboration in the field. Figure 4B further illustrates the partnerships between institutions. Harvard, Michigan, and Chicago stand out as central hubs, with strong collaboration across various institutions. The network highlights numerous partnerships, with leading institutions forming clusters of high research output. This interconnectedness suggests a collaborative research environment, where these institutions lead not only in research production but also in fostering global knowledge exchange.

The distribution of institutions highlights the dominance of institutions like University of Michigan, Harvard University, and Capital Medical University, which consistently lead in publication output. As shown in Figure 4C, these institutions also exhibit the highest number of articles, with University of Michigan and Harvard University contributing the most. The production overtime in Figure 4D further emphasizes the exponential rise in publications from these institutions, particularly after 2005, with Capital Medical University showing a notable increase in recent years. This indicates that although Harvard and Michigan have been leaders in the field for a long time, newer institutions like Capital Medical University are quickly boosting their research output, contributing to the global growth of research in this area (Table 1).

**Table 1:** Top 10 authors on cognition in pediatric OSA (1983–2025).

Rank	Author	h_index	g_index	m_index	TC	NP	PY_start	Articles	Articles fractionalized
1	Gozal, David	41	81	1.708	6,744	81	2002	81	22.10
2	Kheirandish-Gozal, Leila	30	49	1.579	2,764	49	2007	49	11.42
3	Chervin, Ronald D.	18	22	0.692	3,291	22	2000	22	2.22
4	Ni, Xin	9	14	1.125	215	21	2018	21	2.24
5	Horne, Rosemary S. C.	14	20	0.933	900	20	2011	20	3.37
6	Nixon, Gillian M.	14	19	0.933	880	19	2011	19	3.37
7	Xu, Zhifei	6	12	0.75	157	19	2018	19	2.18
8	Redline, Susan	12	16	0.5	1761	16	2002	16	1.50
9	Davey, Margot J.	12	15	0.8	684	15	2011	15	2.39
10	Garetz, Susan L.	12	14	0.48	1986	14	2001	14	1.24

TC, total citations; NP, number of publications; PY_start, year of first publication.

### 3.4 Distributions of authors and co-cited authors

The scholarly landscape of childhood OSA and cognition research from 1983 to 2025 reveals a highly collaborative environment, with 7,409 authors contributing to 1,610 publications. Among these, only 98 authors worked solo, with an average of 5.79 co-authors per paper, indicating the extensive teamwork in this multidisciplinary field (Supplementary Table S1).

**Figure 5:**
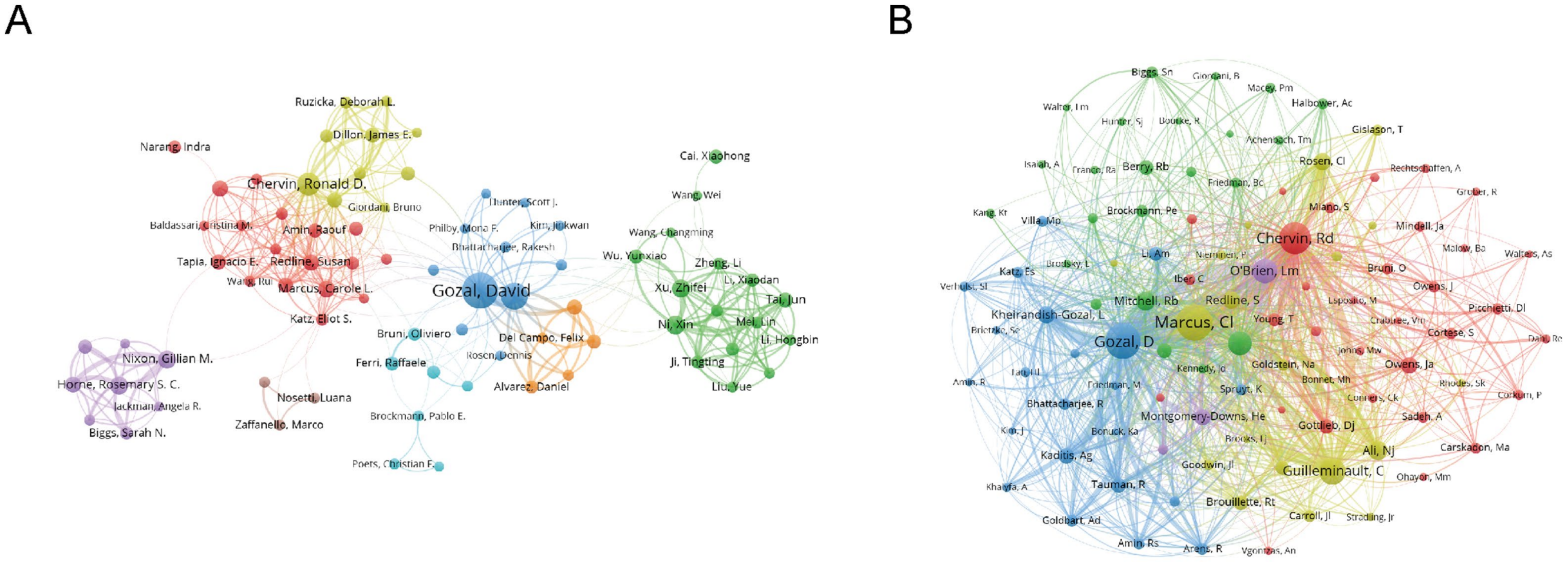
Co-authorship and co-cited authorship networks on cognition in pediatric OSA (1983–2025). **(A)** Co-authorship network: Nodes represent authors, sized by publication count. Colors indicate collaboration clusters. **(B)** Co-cited authorship network: The thickness of the edges between nodes, which are linked by collaborative publications, reflects the number of joint works. Colors indicate clusters that represent research groups.

Figures 5A,B provide further insight into collaboration patterns and author influence. David Gozal, with an h-index of 41 and 81 publications, stands out as the most prolific and influential author in the field, with 6,744 total citations, reflecting his sustained and impactful contributions. Similarly, Leila Kheirandish-Gozal, who has an h-index of 30 and 49 publications, also holds substantial influence in the field. These high h-index scores highlight both productivity and lasting academic impact, as the h-index measures the quantity and quality of an author’s work. The co-cited author network in Figure 5B emphasizes the collective impact of these leading authors. David Gozal, Leila Kheirandish-Gozal, and Ronald Chervin emerge as key figures in this network, linked by a dense web of co-citations, showcasing their pivotal role in advancing research. Their g-index and m-index further reveal that these authors not only publish extensively but also have a large number of highly cited works, reinforcing their central position in the field. The collaborative nature of the research is evident from the low percentage of single-authored papers (1.1%) and the high number of co-authors per publication. The rising trend of international collaborations, reflected in 10.5% international co-authorship, underscores the growing global involvement in this field, particularly among institutions in North America, Europe, and Asia, fostering cross-border academic partnerships that drive progress in the field (Supplementary Table S1).

### 3.5 Journals and co-journals

**Figure 6:**
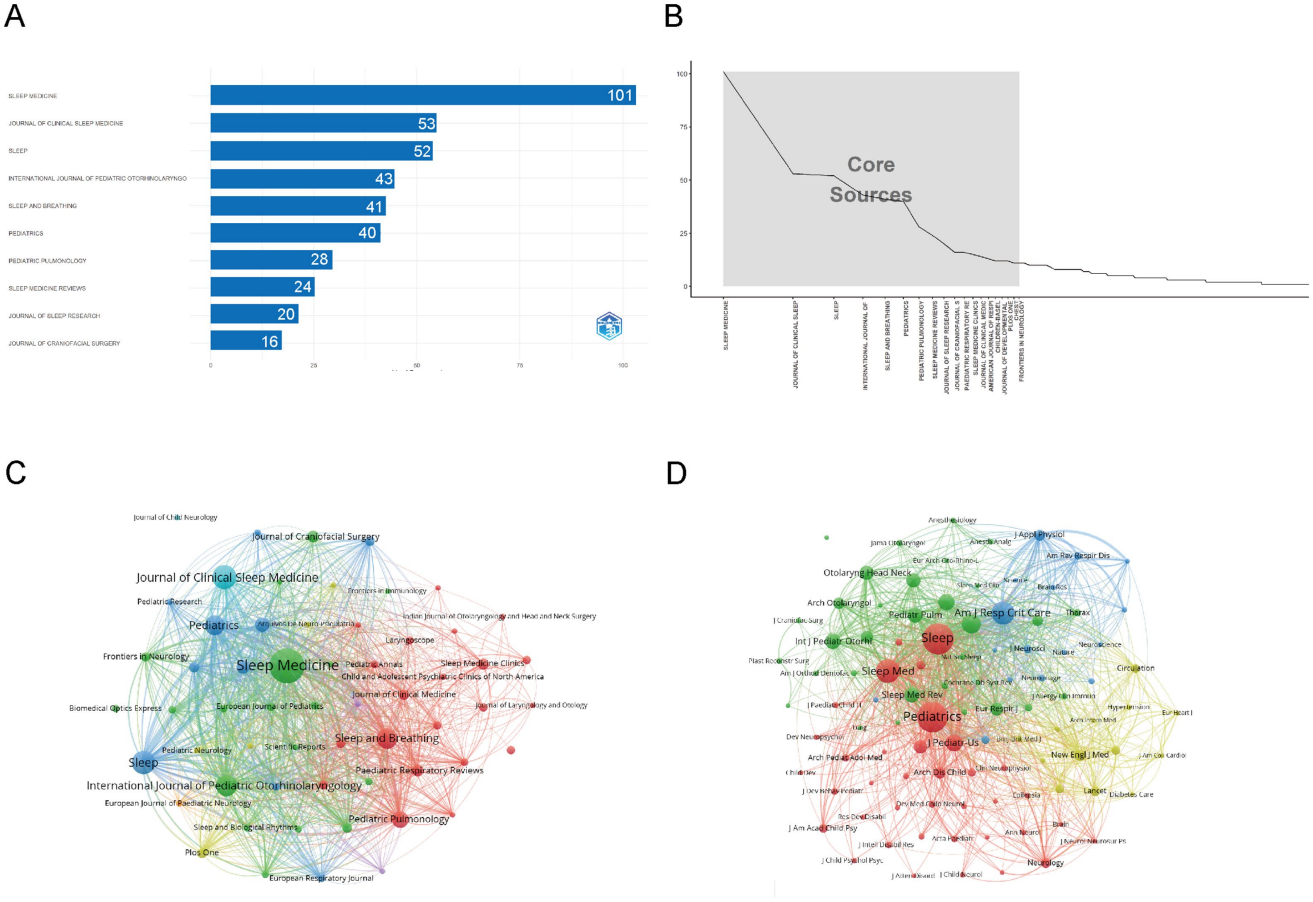
Comprehensive analysis of journals and citation networks related to cognition in pediatric OSA (1983–2025). **(A)** Top journals by publication count: The x-axis represents article numbers, while the y-axis represents journals. **(B)** Bradford’s Law analysis: Recognizes essential journals based on how publications are distributed. **(C)** Journal citation network. Each node represents a journal. Node size is proportional to the number of publications, while links indicate citation relationships between journals. Line thickness reflects the strength of citation connections. Distinct colors (green, blue, red) denote clusters of journals that frequently cite each other, corresponding to thematic areas of research. **(D)** Journal co-citation network: Each node symbolizes a journal, with its size based on how often it is co-cited. The links show co-citation connections, where thicker lines denote stronger co-citation ties. Colors highlight clusters of journals frequently co-cited together.

Figure 6A shows that *Sleep Medicine* is the most influential source in childhood OSA and cognition research, with 101 publications, emphasizing its central role in disseminating high-impact studies. It is followed by *Journal of Clinical Sleep Medicine* (53 articles) and *Sleep* (52 articles), both of which significantly contribute to shaping research in this domain. Table 2 further verifies their academic influence: the journal *Sleep* ranks first with an h-index of 33, thus holding a Q1 JCR ranking; the h-index of *Sleep Medicine* is also 33, while Pediatrics has an annual citation count exceeding 7,000 and is widely published on key topics such as sleep disorders and cognitive impact. These journals’ impact is evident not just in the number of publications but also in their academic prominence and standing.

**Table 2:** Top 10 influential journals on cognition in pediatric OSA (1983–2025).

Rank	Source	h_index	g_index	m_index	TC	NP	PY_start	IF	JCR
1	Sleep	33	52	1.138	4,353	52	1997	4.9	Q1
2	Sleep Medicine	33	64	1.269	4,289	101	2000	3.4	Q2
3	Pediatrics	32	40	1.143	7,367	40	1998	6.4	Q1
4	Sleep Medicine Reviews	19	24	0.826	1,582	24	2003	11.2	Q1
5	Sleep and Breathing	18	33	0.692	1,154	41	2000	2	Q3
6	Journal of Clinical Sleep Medicine	16	28	0.762	864	53	2005	3.5	Q1
7	Pediatric Pulmonology	15	28	0.682	961	28	2004	2.3	Q2
8	International Journal of Pediatric Otorhinolaryngology	13	27	0.464	816	43	1998	1.5	Q3
9	American Journal of Respiratory and Critical Care Medicine	11	13	0.458	1824	13	2002	19.4	Q1
10	Chest	10	11	0.4	767	11	2001	8.6	Q1

IF, impact factor; JCR, journal citation reports.

Figure 6B illustrates the core sources, following Bradford’s Law. The graph highlights *Sleep Medicine* as the dominant journal, followed by *Journal of Clinical Sleep Medicine* and *Sleep*. These core journals, located in the steep decline section, collectively contribute the most articles, indicating their central role in disseminating high-impact research in this field.

As shown in Figure 6C, the analysis reveals a clear concentration of influential sources, with *Sleep Medicine*, *Journal of Clinical Sleep Medicine*, and *Sleep* dominating both publication volume and academic impact. These journals form the core of the research landscape, reflecting their central role in advancing high-impact studies. Figure 6D further emphasizes the academic significance of these core journals, which are frequently cited together, underlining their collaborative relationship in the scientific community.

### 3.6 References and articles

From 1983 to 2025, the bibliographic landscape on childhood OSA and cognition consisted of 1,610 documents gathered from 623 different sources. The dataset was supported by 58,525 cited references, averaging 35.48 citations per document. Original research made up the majority, with 1,443 articles, along with 167 narrative or systematic reviews (Supplementary Table S1).

As shown in Table 3, Gozal D (1998) and Marcus CL (2013) lead with 164 and 136 local citations, respectively. Gozal’s paper was the first to show that sleep-disordered breathing (SDB) affects children’s academic performance, while Marcus’s study demonstrated notable improvements in behavior, symptoms, and quality of life, although cognitive gains were limited. Beebe DW’s (2002) prefrontal-cortex model (122 local citations) and Halbower AC’s (2006) neuroimaging study (77 local citations) further solidified the connection between nocturnal hypoxia and daytime neurobehavioral deficits. Together, these four highly-cited works outline the three main research themes—epidemiology, intervention, and neurobiology—around which contemporary research on childhood OSA and cognition is structured.

**Table 3:** Top 20 most local cited publications based on bibliometrix analysis (1983–2025).

Rank	First author	Year	Journal	Paper	DOI	Local citations	Global citations	LC/GC ratio (%)	Normalized local citations	Normalized global citations
1	Gozal D.	1998	Pediatrics	Sleep-disordered breathing and school performance in children	10.1542/peds.102.3.616	164	801	20.47	5.96	4.57
2	Marcus C. L.	2013	New Engl J Med	A randomized trial of adenotonsillectomy for childhood sleep apnea	10.1056/NEJMoa1215881	136	964	14.11	40.62	21.09
3	Beebe D. W.	2002	J Sleep res	Obstructive sleep apnea and the prefrontal cortex: toward a comprehensive model linking nocturnal upper airway obstruction to daytime cognitive and behavioral deficits	10.1046/j.1365-2869.2002.00289.x	122	663	18.40	4.92	3.09
4	Marcus C. L.	2012	Pediatrics	Diagnosis and management of childhood obstructive sleep apnea syndrome	10.1542/peds.2012-1672	109	1,149	9.49	16.24	17.93
5	O’brien L. M.	2004	Pediatrics	Neurobehavioral Implications of Habitual Snoring in Children	10.1542/peds.114.1.44	104	374	27.81	6.38	3.53
6	Chervin R. D.	2000	Sleep Med	Pediatric sleep questionnaire (PSQ): validity and reliability of scales for sleep-disordered breathing, snoring, sleepiness, and behavioral problems	10.1016/S1389-9457(99)00009-X	101	1,021	9.89	11.43	6.21
7	Gozal D.	2001	Pediatrics	Snoring during early childhood and academic performance at ages thirteen to fourteen years	10.1542/peds.107.6.1394	95	300	31.67	5.97	2.92
8	O’brien L. M.	2003	Pediatrics	Sleep- and neurobehavioral characteristics of 5- to 7-year-old children with parent-reported symptoms of attention-deficit/hyperactivity disorder	10.1542/peds.111.3.554	89	394	22.59	4.58	3.87
9	Beebe D. W.	2006	Sleep	Neurobehavioral morbidity associated with disordered breathing during sleep in children: a comprehensive review	10.1093/sleep/29.9.1115	89	320	27.81	6.24	3.65
10	Chervin R. D.	2006	Pediatrics	Sleep-disordered breathing, behavior, and cognition in children before and after adenotonsillectomy	10.1542/peds.2005-1837	84	401	20.95	5.89	4.57
11	Halbower A. C.	2006	Plos Med	Childhood obstructive sleep apnea associates with neuropsychological deficits and neuronal brain injury	10.1371/journal.pmed.0030301	77	254	30.31	5.39	2.89
12	Gottlieb D. J.	2004	J Pediatrics	Sleep-disordered breathing symptoms are associated with poorer cognitive function in 5-year-old children	10.1016/j.jpeds.2004.05.039	76	191	39.79	4.66	1.80
13	Chervin R. D.	2002	Pediatrics	Inattention, hyperactivity, and symptoms of sleep-disordered breathing	10.1542/peds.109.3.449	72	382	18.85	2.90	1.78
14	Friedman B. C.	2003	Sleep	Adenotonsillectomy improves neurocognitive function in children with obstructive sleep apnea syndrome	10.1093/sleep/26.8.999	68	164	41.46	3.50	1.61
15	Chervin R. D.	1997	Sleep	Symptoms of sleep disorders, inattention, and hyperactivity in children	10.1093/sleep/20.12.1185	63	404	15.59	4.00	2.77
16	Kennedy J. D.	2004	Pediatr Pulm	Reduced neurocognition in children who snore	10.1002/ppul.10453	63	149	42.28	3.86	1.41
17	Montgomery-Downs H. E.	2005	Eur Respir J	Cognition, sleep and respiration in at-risk children treated for obstructive sleep apnoea	10.1183/09031936.05.00082904	57	139	41.01	6.92	1.76
18	Hunter S. J.	2016	Am J Resp Crit Care	Effect of sleep-disordered breathing severity on cognitive performance measures in a large community cohort of young school-aged children	10.1164/rccm.201510-2099OC	53	176	30.11	14.64	3.57
19	Urschitz M. S.	2003	Am J Resp Crit Care	Snoring, intermittent hypoxia and academic performance in primary school children	10.1164/rccm.200212-1397OC	46	173	26.59	2.37	1.70
20	Bourke R.	2011	Sleep Med	Cognitive and academic functions are impaired in children with all severities of sleep-disordered breathing	10.1016/j.sleep.2010.11.010	46	162	28.40	9.79	3.87
21	Gottlieb D. J.	2003	Pediatrics	Symptoms of sleep-disordered breathing in 5-year-old children are associated with sleepiness and problem behaviors	10.1542/peds.112.4.870	45	186	24.19	2.31	1.83
22	Melendres M. C. S.	2004	Pediatrics	Daytime sleepiness and hyperactivity in children with suspected sleep-disordered breathing	10.1542/peds.2004-0730	44	329	13.37	2.70	3.11
23	Stradling J. R.	1990	Lancet	Effect of adenotonsillectomy on nocturnal hypoxaemia, sleep disturbance, and symptoms in snoring children	10.1016/0140-6736(90)90068-G	42	346	12.14	1.00	1.00
24	Rosen C. L.	2004	Pediatrics	Increased behavioral morbidity in school-aged children with sleep-disordered breathing	10.1542/peds.2004-0103	41	215	19.07	2.51	2.03
25	Gozal D.	2007	Am J Resp Crit Care	C-reactive protein, obstructive sleep apnea, and cognitive dysfunction in school-aged children	10.1164/rccm.200610-1519OC	41	200	20.50	5.61	2.12

DOI, digital object identifier.

**Figure 7:**
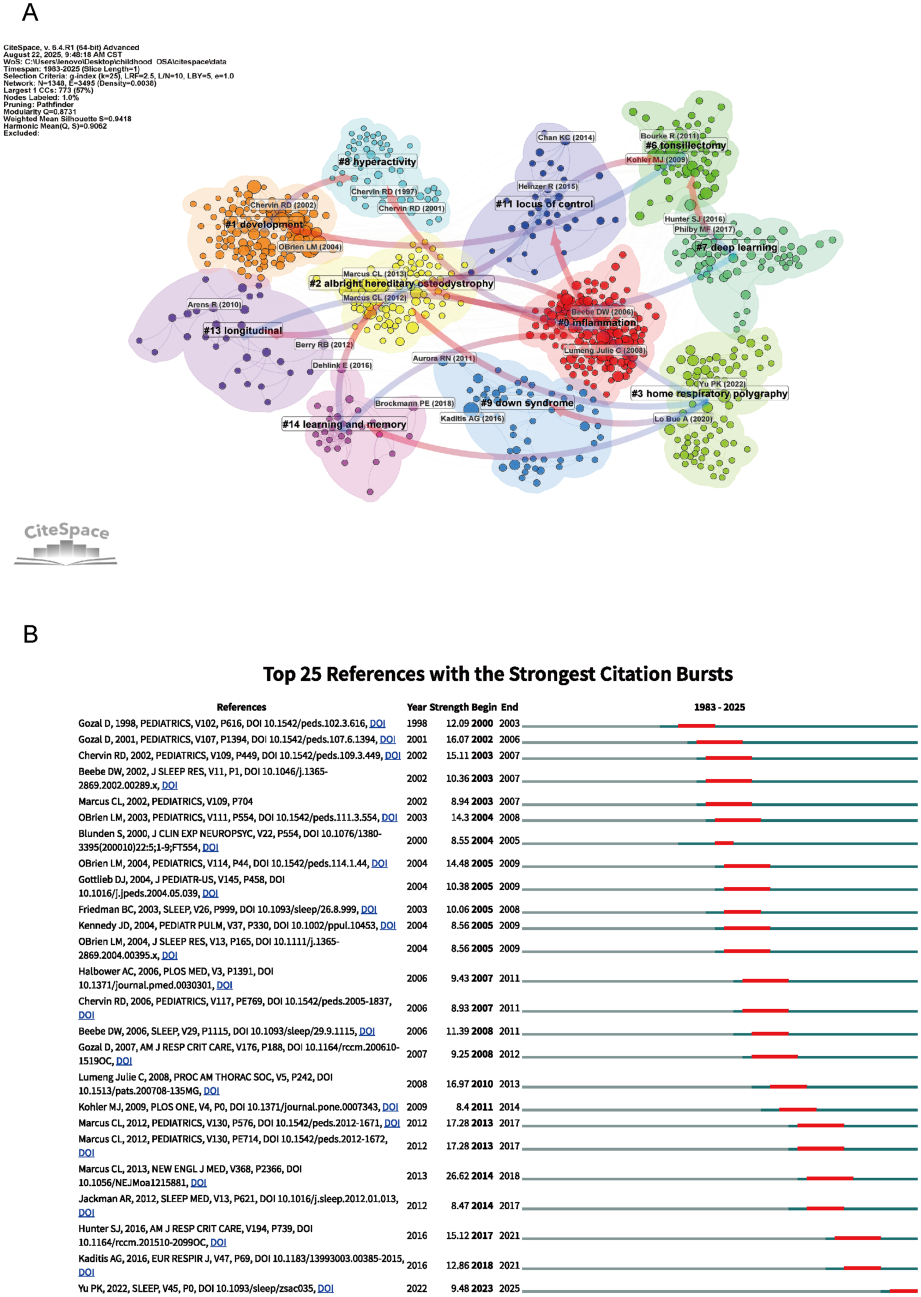
Network visualization of references and top 25 references with strongest citation bursts on cognition in pediatric OSA (1983–2025). **(A)** Clustered co-citation network based on keywords: Nodes sized by citation frequency are clustered by modularity with keywords; edge thickness shows co-citation strength, and colors indicate thematic differences. **(B)** The burst timeline highlights periods of increased attention, with red segments marking when each reference drew heightened focus.

Figure 7A reveals a detailed co-citation clustering map of childhood OSA research, highlighting distinct themes with a focus on cognitive, clinical, and developmental aspects. Cluster 0 (“Inflammation”) stands out as a critical factor in the pathophysiology of childhood OSA. Research in this cluster underscores the role of inflammation in exacerbating sleep apnea symptoms and its contribution to cognitive deficits (17). Inflammatory markers have been linked to neurocognitive impairments in children with OSA, suggesting that addressing inflammation may improve both cognitive function and sleep quality, providing insights for potential therapeutic targets (18). The themes reflected in Clusters 1 (“Development”), 7 (“Deep Learning”), 14 (“Learning and Memory”), and 11 (“Locus of Control”) bring together perspectives on how cognitive, behavioral, and psychological aspects interact in the context of OSA. Studies grouped under these clusters point to the ways in which OSA can interfere with both cognitive growth and developmental processes, with memory, learning ability, and attention span being particularly affected (19). Cognitive impairments often coexist with behavioral disturbances such as hyperactivity, potentially intensifying the overall neurodevelopmental burden. In recent research, deep learning models have been applied to disentangle these multidimensional associations (6, 20). A child’s psychological profile, particularly their belief in personal control over health, plays a role in treatment compliance and therapeutic results. Therefore, interventions need to consider these mental dimensions (21). Cluster 9 (“Down Syndrome”) and Cluster 8 (“Hyperactivity”) examine groups with unique vulnerabilities to OSA. Among children with Down syndrome, the likelihood of experiencing OSA is elevated, and this condition has been associated with delays in both cognitive and developmental domains (22). The Hyperactivity cluster examines how OSA is associated with behavioral disorders such as ADHD, showing the significant impact of sleep apnea on children’s attention and behavior. Clusters 6 (“Tonsillectomy”), 2 (“Albright hereditary osteodystrophy”), and 3 (“Home Respiratory Polysomnography”) focus on clinical approaches and diagnostic advances. Procedures such as tonsillectomy have shown effectiveness in managing OSA, particularly among pediatric patients with anatomical obstructions like enlarged tonsils (23). The use of Home Respiratory Polysomnography has improved diagnostic accuracy while making sleep assessment more accessible in non-hospital settings. Research on Albright hereditary osteodystrophy underscores the genetic contribution to OSA, offering insights into how comorbid conditions may exacerbate both sleep-related symptoms and associated cognitive impairments. By linking behavioral, cognitive, clinical, and technological findings, the clustering analysis offers a multidimensional perspective on pediatric OSA and informs strategies for its clinical management.

Figure 7B shows the 25 most influential references in pediatric obstructive sleep apnea research, ranked by their citation burst strength between 1983 and 2025. Citation burst strength reflects periods of increased academic attention to specific studies. For instance, the study by Hunter et al. (2016) (24) stands out with a citation burst strength of 15.12 in 2017, reflecting its significant impact on understanding the cognitive consequences of sleep-disordered breathing (SDB) in children. Similarly, Yu et al. (2022) (25) had a citation burst of 9.48 in 2022, reflecting more attention to the neurobehavioral effects of mild sleep-disordered breathing, especially in children who snore. The guideline by Kaditis et al. (26) showed a burst strength of 12.86 in 2018, pointing to its importance in developing clinical strategies for pediatric OSA. These citation bursts mark major research advances and illustrate how studies on sleep-disordered breathing in children have expanded understanding of its cognitive effects.

### 3.7 Keywords analysis

The keyword analysis reveals thematic breadth and evolving research focus within the field of childhood OSA and cognition. A total of 7,857 Keywords Plus and 2,892 Author Keywords were identified across 1,610 publications (Supplementary Table S1), supporting comprehensive indexing and thematic mapping. The keyword corpus forms the foundation for subsequent co-word and cluster analyses, enabling deeper insight into how key concepts have evolved across time in response to clinical and technological advances in pediatric sleep research.

**Figure 8:**
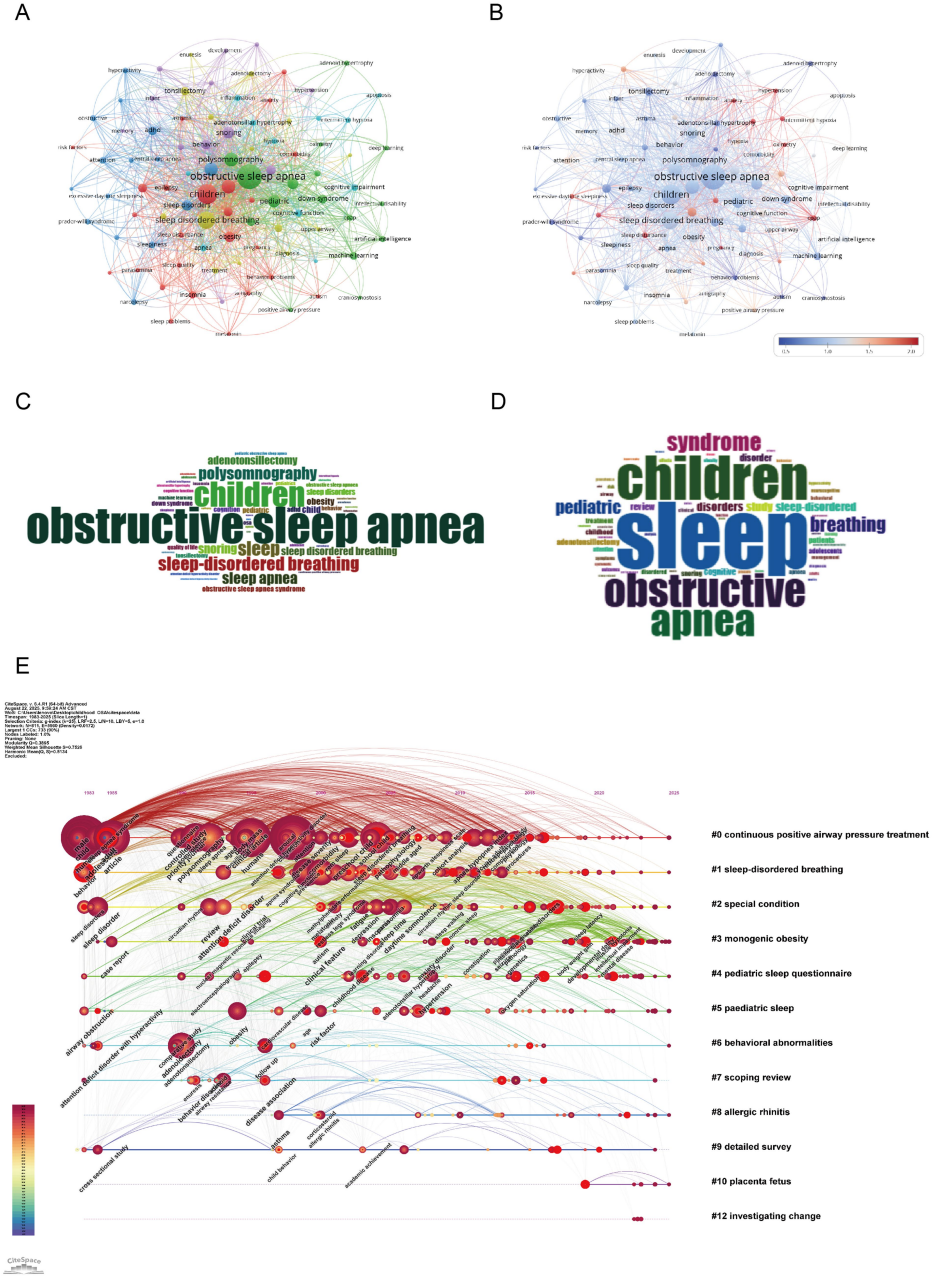
Keywords mapping of cognition in pediatric OSA **(A)** Keywords co-occurrence network: Nodes (keywords) are sized by frequency; edges reflect co-occurrence strength, and colors show modularity-based thematic clusters. **(B)** Keyword centrality network. Each node corresponds to a keyword, with size reflecting frequency and color (redder hues) indicating higher betweenness centrality. Edges show co-occurrence links. The visualization emphasizes key bridging terms across clusters. **(C)** Author keywords word cloud: Word size reflects frequency, highlighting prevalent author-assigned terms. **(D)** Title keywords word cloud: Word size indicates frequency, emphasizing key title-derived themes. **(E)** Keyword timeline visualization. The horizontal axis shows publication years, with each line representing a thematic cluster labeled by a key term. Nodes within clusters indicate keywords, sized by frequency and linked via co-occurrence. Their position marks the first year of appearance, illustrating topic emergence, continuity, and development over time.

As shown in Figures 8A–D, the central theme in childhood OSA research remains “obstructive sleep apnea” (514 occurrences, total link strength = 1,104), “children” (311 occurrences, total link strength = 799), and “sleep disordered breathing” (209 occurrences, total link strength = 541). These keywords confirm the centrality of pediatric sleep-disordered breathing in current research discourse. Several clinical diagnostic terms also rank highly, including “sleep” (137 occurrences, total link strength = 380) and “polysomnography” (126 occurrences, total link strength = 356), reflecting continued reliance on objective sleep measures (27). “Adenotonsillectomy” (81 occurrences, total link strength = 205) and “snoring” (91 occurrences, total link strength = 277) further reinforce the clinical intervention focus, particularly for surgical approaches (23, 28). Importantly, the field shows increasing emphasis on cognitive and behavioral dimensions. Terms such as “cognition” (56 occurrences, total link strength = 166), “behavior” (55 occurrences, total link strength = 179), and “ADHD” (49 occurrences, total link strength = 110) illustrate the rising concern over OSA-related neurobehavioral effects (19). The term “attention deficit hyperactivity disorder” (46 occurrences, total link strength = 118) further supports this association. Keywords like “obesity” (67 occurrences, total link strength = 165) and “Down syndrome” (47 occurrences, total link strength = 94), both related to comorbidities, indicate a focus on at-risk pediatric groups (22). The keyword “machine learning” (35 occurrences, total link strength = 66) reflects growing use of computational tools in diagnosis and prediction (6). The keyword data show that research, though rooted in pediatric respiratory diagnostics, is extending into cognitive, behavioral, and technological domains. Neurodevelopmental and traditional clinical terms appear together, pointing to increased interdisciplinary engagement. These findings help outline potential directions for future research. The overlap among OSA, cognitive function, and personalized diagnostics is likely to remain a key area of interest.

Figure 8E shows a keyword timeline map depicting how major research topics in childhood OSA and cognition have developed over time and formed thematic clusters. The map reveals both long-term patterns and more recent keyword appearances, offering perspective on nearly forty years of research progress. Cluster #1 (“sleep-disordered breathing”) stands out as the most persistent, covering the period from the late 1990s to the 2010s. This indicates continued attention to childhood sleep-related breathing issues. Likewise, Cluster #0 (“continuous positive airway pressure treatment”) also spans many years, reflecting the lasting role of CPAP as the main treatment for moderate to severe OSA (26). This cluster includes terms like attention-deficit disorder, executive dysfunction, and behavioral intervention, pointing to increasing concern about OSA’s neurodevelopmental and psychosocial effect. Newer clusters, including Cluster #7 (“scoping review”), Cluster #9 (“detailed survey”), and Cluster #12 (“investigating change”), suggest a shift toward evidence-based synthesis, policy direction, and long-term follow-up. Cluster #2 (“special condition”) and Cluster #8 (“allergic rhinitis”) center on comorbid or high-risk groups, emphasizing the demand for individualized approaches in diagnosis and treatment (29). Overall, this timeline shows a research field that has progressed from basic clinical concerns to include interdisciplinary topics such as cognition, comorbidity, and innovative study designs. The coexistence of established and emerging clusters indicates a broadening research scope, especially in studies focused on development, behavior, and specific populations.

## 4 Discussion

This analysis offers a comprehensive bibliometric and scientometric perspective on research related to pediatric OSA and cognition, revealing the field’s wide-ranging and developing focus. Our findings demonstrate a steady expansion of publications, with increasing attention to neurocognitive outcomes, mechanistic pathways, and emerging applications of artificial intelligence. These results highlight not only the clinical relevance of OSA-related cognitive impairments but also the shifting research priorities from descriptive epidemiology toward mechanistic insights and precision-based management. Building on these observations, the following discussion addresses key research highlights, current limitations, and future directions.

### 4.1 Research highlights

The analysis of keyword and reference clustering highlights the main areas of interest among researchers. Based on these findings, we outline several significant research hotspots, including cognitive and behavioral effects of pediatric OSA, mechanisms: hypoxia, inflammation, and comorbidities, treatment approaches: surgical and non-surgical, innovations in diagnosis and personalized treatment.

#### 4.1.1 Cognitive and behavioral effects of pediatric OSA

In children, maternal sleep disturbances during pregnancy are correlated with poorer cognitive outcomes, suggesting that sleep-related issues may affect cognitive development early in life (30). Children with moderate to severe OSA show more pronounced impairments in cognitive tests compared to healthy controls, particularly in areas of attention and memory. These impairments lead to difficulties in classroom learning, social interactions, and behavior regulation (31).

##### 4.1.1.1 Cognitive deficits and functional implications in pediatric OSA

Around 60% of children with OSA exhibit deficits in attention, working memory, and episodic memory, which are closely linked to poor sleep quality (31, 32). Standardized tests confirm these impairments, which also affect executive functions (33, 34). Children who have moderate or severe OSA tend to struggle more with tasks that need planning and control than other children (35). Co-occurring anxiety may further exacerbate these impairments (32). Because of these thinking problems, children with OSA may find it hard to learn, get along with others, or behave well. For example, distractibility and slower processing speed can hinder task completion and academic performance. A recent study by Wu et al.(2024) (2) used the Attention Network Test (ANT) along with event-related potentials (ERP) and time-frequency analysis to evaluate attention in children with OSA. The study found that the attention system did not work well, especially the part that helps control actions and deal with conflicts (2). Children with OSA often experience memory deficits that affect both working memory and long-term memory, making it harder for them to retain and recall information. These issues can disrupt learning, creating difficulties in keeping up with the school curriculum. Additionally, struggles with remembering social cues and past interactions can impact peer relationships and social integration. A study by Halbower et al. (36) found that kids with OSA did not do well on IQ tests. They also had problems with memory and speaking skills. Cognitive deficits in children with OSA are associated with changes in neuronal metabolites in the hippocampus and frontal cortex, both of which are crucial for memory and executive function. When this happens, some kids become too active, act without thinking, or find it hard to control their feelings. These behaviors often cause trouble at school. Teachers may misunderstand them, and sometimes they are thought to have ADHD by mistake. Socially, these challenges can make it harder for children to build and maintain friendships, leading to feelings of isolation and lower self-esteem (37). A study by Beebe et al. (37) found that children with OSA exhibited significant impairments in behavior regulation and some aspects of attention and executive functioning. This was seen most clearly in how they behaved. It shows how OSA can affect both their feelings and how they deal with others.

##### 4.1.1.2 Sleep quality and cognitive performance

When children do not sleep well — like sleeping too little or waking up a lot during the night — it becomes harder for them to focus, remember things, or plan their actions. These skills are important for how their thinking develops over time. OSA in children often leads to excessive daytime sleepiness and disrupted nocturnal sleep, which in turn impairs sustained attention and learning efficiency (38). Furthermore, lower sleep quality and shorter sleep duration are significantly associated with declines in task accuracy, response speed, and working memory performance in children with OSA (39).

##### 4.1.1.3 Comorbidities and neurocognitive impacts

The interaction between children, OSA and ADHD is characterized by a significant association, with ADHD increasing the risk of developing OSA and vice versa. This bidirectional relationship is potentially linked by inflammation in specific brain regions (40). Children with ADHD have a high prevalence of OSA, with studies showing that 23.4 to 50% of children with ADHD are diagnosed with OSA, and a significant portion exhibit symptoms like snoring (41, 42).

The severity of OSA in children with ADHD correlates with increased ADHD symptoms, including inattention, hyperactivity, and behavioral disorders, suggesting that OSA may exacerbate these symptoms (43). The co-occurrence of ADHD and OSA may produce a synergistic burden, particularly impairing attentional focus, short-term memory retention, and planning ability. Both ADHD and OSA independently contribute to attention deficits. When co-occurring, the impact is magnified. OSA-related sleep fragmentation leads to daytime sleepiness and impaired attention, while ADHD is characterized by inattention and impulsivity. This combination results in more pronounced difficulties in sustaining attention and completing tasks. Children with OSA also exhibit altered neurotransmitter levels and higher serum leptin, which correlate with ADHD symptoms (44). Additionally, there is a genetic correlation between ADHD and OSA, with ADHD increasing the risk of OSA (OR 1.09, 95% CI: 1.01–1.17) (45). In children with Down syndrome, OSA negatively affects neurocognitive development, impacting 55–90% of this population (46).

The presence of both ADHD and OSA in children requires a thorough approach to diagnosis and treatment. Clinicians should evaluate both conditions when children present with cognitive and behavioral issues. Diagnostic tools, such as the Conners’ Rating Scales for ADHD and PSG for OSA, can help with assessment. Treatment plans should tackle both conditions at once. For example, CPAP therapy for OSA may improve sleep quality and reduce some of the cognitive issues linked to both OSA and ADHD. Additionally, behavioral interventions aimed at ADHD symptoms can also provide benefits.

#### 4.1.2 Mechanisms: hypoxia, inflammation, and comorbidities

Current evidence suggests that pediatric OSA disrupts cognitive development through several interrelated biological pathways: in which sleep fragmentation deprives the developing brain of restorative function of rapid eye movement sleep (REM) and slow-wave Sleep (SWS), while intermittent hypoxemia causes oxidation, inflammation, and vascular stress (47). These disturbances jointly affect neural circuits that govern attention, memory, and higher-order executive processes—domains that are particularly fragile during childhood maturation.

##### 4.1.2.1 Sleep fragmentation and its cognitive consequences

One key pathway is sleep fragmentation accompanied by changes in sleep stage composition. Recurrent arousals interrupt sleep continuity and disturb the balance between REM sleep and SWS (48). Disturbance of REM is of special concern, as this stage fosters hippocampal–cortical dialog that underlies memory integration and the regulation of affect (49). When REM sleep is fragmented, it is often associated with poorer declarative memory and greater emotional volatility in children (50). Reduced SWS poses a similar risk: it weakens slow oscillation–spindle synchrony and hampers hippocampo-cortical replay, both of which are crucial for consolidating declarative memory and supporting executive control (50, 51). Furthermore, because SWS facilitates the release of neurotrophic factors and growth hormone, its reduction may compromise structural brain growth (52). Among cortical regions, the prefrontal cortex (PFC), central to attentional control and decision-making, appears especially vulnerable. Pediatric imaging studies consistently document altered PFC activation patterns and structural deviations in children with OSA (53).

##### 4.1.2.2 Intermittent hypoxia, inflammation, and neurochemical changes

A second critical mechanism of injury arises from intermittent hypoxemia (54). Clinical studies show that lower nocturnal oxygen saturation (SpO₂) is linked to poorer immediate recall, reduced vigilance, and slower processing speed in children with OSA, directly connecting intermittent hypoxia to decreased neurocognitive efficiency (33, 34). Hypoxic episodes activate the Nuclear Factor kappa-light-chain-enhancer of activated B cells (NF-κB) pathway and provoke an increase in pro-inflammatory cytokines, including interleukin-6 (IL-6) and tumor necrosis factor-*α* (TNF-α) (17). Once released, these inflammatory mediators can either penetrate the blood–brain barrier or stimulate resident microglia, setting the stage for sustained neuroinflammation (55). The downstream effects include impaired synaptic remodeling and a reduction in brain-derived neurotrophic factor, changes that weaken neural plasticity and may divert normal cortical development (56).

Pediatric obstructive sleep apnea (OSA) is associated with dysregulation of the autonomic nervous system and hypothalamic–pituitary–adrenal (HPA) axis, both of which contribute to cognitive and behavioral impairments (57). OSA has been shown to elevate sympathetic nervous system activity and reduce parasympathetic tone, with intermittent hypoxia further enhancing sympathetic drive and triggering the release of catecholamines, such as norepinephrine and adrenaline, as corroborated by clinical studies (57). Additionally, repeated arousals and hypoxemia lead to the elevation of stress hormones, particularly cortisol, resulting in long-term alterations in HPA axis function (58). Chronic exposure to cortisol negatively impacts synaptic stability, especially in the PFC and hippocampus—regions crucial for memory, attention, and the regulation of behavior (59).

Neurotransmitter imbalances also contribute to cognitive deficits associated with OSA Increased extracellular glutamate levels are implicated in excitotoxicity, particularly within the hippocampal circuits, leading to neuronal damage (60). Furthermore, a reduction in gamma-aminobutyric acid (GABA)ergic tone has been observed, which may disrupt synchrony between brain regions, thereby impairing cognitive processes and behavioral control (60). Disruption of the cholinergic system, which plays a key role in attention regulation and REM sleep, has also been noted in OSA, potentially interfering with these functions (61). In addition, dopaminergic imbalances, particularly within the prefrontal-striatal circuits, may affect impulse control and emotional regulation (62).

Cerebrovascular alterations appear to intensify the neural consequences of pediatric OSA (18). When endothelial function is compromised and nitric oxide availability declines, neurovascular coupling becomes inefficient, restricting the normal rise in cerebral blood flow that accompanies neuronal activity (63). Pediatric neuroimaging evidence supports this mechanism, showing that children with OSA often have disrupted white-matter structure and unusual cortical thickness in the prefrontal and hippocampal regions. These findings reflect the vascular and metabolic stress that occurs during critical stages of brain development (64–66). The convergence of these processes on prefrontal–hippocampal circuits explains the characteristic profile of attention deficits, impaired memory, and behavioral disturbances observed in children with OSA. Importantly, the timing of exposure during sensitive neurodevelopmental windows may determine whether these deficits resolve after treatment or persist into adolescence (67). Additionally, studies have shown that children with OSA exhibit deficits in learning and memory, even after undergoing AT, suggesting irreversible hippocampal damage due to early-life intermittent hypoxia (68).

##### 4.1.2.3 Mitochondrial dysfunction and oxidative stress

Oxidative stress and mitochondrial dysfunction play pivotal roles in the cognitive impairments observed in children with OSA. During sleep, repeated episodes of oxygen desaturation and reoxygenation disrupt mitochondrial function, leading to an accumulation of reactive oxygen species (ROS) (69). These mitochondrial alterations not only impair energy production but also exacerbate neuronal injury (69). Regions such as the hippocampus and PFC, which are essential for memory and executive function, are particularly vulnerable to oxidative damage due to their high metabolic activity (53). Beyond affecting neuronal survival, mitochondrial dysfunction impedes synaptic plasticity, contributing to deficits in cognitive abilities like learning and memory (70). Moreover, oxidative stress stimulates microglia, resulting in chronic neuroinflammation that further aggravates neuronal dysfunction (71). The continuous cycle of oxidative damage and mitochondrial impairment creates a feedback loop that disrupts brain development in children with OSA (36, 72). Targeting oxidative stress and mitochondrial dysfunction may offer promising therapeutic avenues for alleviating the cognitive deficits seen in these children.

#### 4.1.3 Treatment approaches: surgical and non-surgical

AT is considered the first-line therapy for pediatric obstructive sleep-disordered breathing. The Childhood Adenotonsillectomy Trial (CHAT; n = 464) assessed the NEPSY attention/executive function at 7 months as the primary neurocognitive outcome. No significant difference was observed between the surgical and observation groups (+7.1 ± 13.9 vs. +5.1 ± 13.4, *p* = 0.16). Nonetheless, surgery led to a significantly higher rate of polysomnographic normalization (79% vs. 46%) (23), along with notable improvements in behavior and quality of life. A randomized controlled trial by Waters et al. (28) examined preschool children with mild OSA. After 12 months, no significant improvement in global IQ was found in the AT group compared to the waitlist control. However, children in the surgery group showed improvements in sleep quality and parent-reported behavior. Similarly, the Pediatric Adenotonsillectomy Trial (PATS) found no improvement in attention or executive function in children with mild disease. However, secondary outcomes—including behavior, symptoms, and blood pressure—showed significant improvement. Progression of apnea–hypopnea index (AHI) beyond 3 events/h was far less common after surgery (1.3% vs. 13.2%). Serious adverse surgical events occurred in about 2.7% of cases (73). A prospective study by Shan et al. (74) found that AT significantly improved attention and quality of life, and also reduced anxiety in children with OSA. These findings suggest that while cognitive functions may not fully normalize, certain aspects of cognitive and emotional well-being may improve post-surgery.

CPAP is an alternative for patients with residual disease or who are unsuitable for surgery. In a prospective pre–post study (n = 52), three months of CPAP significantly improved attention, daytime sleepiness, and behavioral scores (all *p* < 0.001) (75). Nonetheless, adherence was poor, averaging only 170 ± 145 min per night. Improvement in sleepiness correlated with usage duration (*r* = 0.411). A meta-analysis by Jiang et al. (76) found that CPAP therapy modestly improved attention and executive function in individuals with OSA. Notably, the benefits were more pronounced in individuals with good adherence (≥4 h/night) and those undergoing short-term treatment (<8 weeks). Anti-inflammatory therapy provides a potential adjunctive option. Montelukast for 16 weeks reduced AHI from 9.2 ± 4.1 to 4.2 ± 2.8 events/h, with clinical benefit in 71.4% of children compared with 6.9% in the placebo group (77).

Both AT and CPAP therapy can improve cognitive functions in children with OSA, though the degree of recovery varies depending on factors like treatment adherence, duration, and individual patient characteristics. AT may not fully restore cognitive abilities, but it can enhance attention and emotional regulation. CPAP therapy has the potential to improve cognitive outcomes, especially when adherence is maintained and the treatment is customized for each child. These findings emphasize the importance of a personalized approach that considers each patient’s unique needs and characteristics to optimize cognitive outcomes in pediatric OSA.

#### 4.1.4 Integrating diagnostic innovations with cognitive outcomes

ML is changing how doctors screen and sort children with OSA. Some models use ECG, oxygen levels, and sound signals to find OSA and guess how serious it is, even without full sleep studies (5). In children, deep learning tools that look at overnight oxygen data and work through cloud systems have already been used at home or in clinics (6). Some recent research built ML models using common signs and symptoms to help diagnose OSA in children. These may offer a simpler option than full sleep tests. For example, Qin et al. (78) created an ML screening model based on children’s clinical characteristics, achieving an average area under the curve (AUC) of 0.73 for AHI ≥ 5 and 0.78 for AHI ≥ 10. This model showed promising results compared to traditional PSG questionnaire-based approaches, offering a potentially more cost-effective and accessible diagnostic option. But to know if it really helps in practice, more cost studies and tests in different hospitals are still needed. Such approaches could enable the early detection of high-risk patients and support timely referral, once validated for routine clinical use.

AI tools, especially deep learning systems, have been useful in spotting OSA by looking at signals such as oxygen levels and heart activity (79). But using them in real clinics is still being studied. AI is also being used to guide treatment by grouping patients based on specific traits. However, doctors are still testing how well this works in everyday practice. By identifying patient subgroups most likely to benefit from AT or CPAP, AI-guided approaches could help reduce unnecessary surgery, improve adherence, and increase treatment success (7), though real-world implementation requires further validation.

Finding OSA early with the help of ML and AI may let doctors act sooner. This could help prevent some thinking or learning problems. Still, more studies are needed to see if this works in daily care. By identifying high-risk patients early, clinicians can start appropriate treatments to prevent or reduce cognitive deficits, ultimately enhancing the overall quality of life for affected children (79). Integrating diagnostic advances such as ML and AI into clinical practice holds great potential for improving the diagnosis and treatment of pediatric OSA. Since these tools can spot high-risk cases early and more clearly, they may help doctors choose better treatments that protect thinking skills in children with OSA. More research is still needed to make results better and improve how we care for kids with OSA (8).

### 4.2 Prospects for the future

Future research on pediatric obstructive sleep apnea (OSA) and cognition should focus more on clinical application. Large-scale, long-term studies are essential to determine which cognitive deficits improve with treatment and which persist, helping to guide when and how interventions should be applied. Including neuroimaging, cognitive tests, and biomarkers in clinical cohorts will provide objective indicators for prognosis and follow-up care. Research should also work on validating ML and AI models across a range of pediatric populations and integrating them into primary care. Ensuring these models are validated across diverse groups and integrated into primary care is crucial before they can be widely adopted. Cost-effectiveness studies and simplified workflows will be necessary to assess their practicality, especially in community and low-resource settings. Precision medicine, potentially guided by AI-driven phenotyping, could help target treatments more effectively, and future research should also look into ways to improve therapy adherence and outcomes. Enhancing adherence, particularly for CPAP, through digital monitoring, family education, and behavioral support will be key to achieving lasting cognitive and developmental benefits. Special populations, such as children with Down syndrome, obesity, or ADHD, need personalized care plans, as they face compounded risks of OSA and neurocognitive issues. Collaboration among pediatricians, sleep specialists, neurologists, and educators will be essential to improving both medical outcomes and social or academic functioning. For instance, a typical multi-site study design may involve pediatric sleep specialists identifying and treating OSA cases (e.g., with AT or CPAP), while neuroimaging researchers conduct high-resolution T1 and diffusion tensor imaging (DTI) scans to look for changes in brain structure, and psychologists administer standardized cognitive tests such as the Wechsler Intelligence Scale for Children (WISC) or similar tools (e.g., Das–Naglieri Cognitive Assessment System (DN-CAS)) to assess functional outcomes (80–82). One study by Mei et al. (64) found that children with severe OSA had weaker white matter in the front part of the brain. This brain change was closely linked to problems with working memory and attention. Bringing together clinical findings, brain scans, and behavior test results helps researchers better understand how OSA affects brain development. These combined insights also support decisions about when and how to treat the condition.

Another collaborative model involves partnerships between Otolaryngologists and child behavioral teams. Researches have shown that children with OSA often show emotional outbursts, high activity levels, and behavior issues, even in children with no overt neurological comorbidities (83). Tools like the Child Behavior Checklist (CBCL) and Behavior Assessment System for Children – 2nd Edition (BASC-2) are often used before and after treatment to track changes in behavior. However, few studies have examined how adding parent training or therapy might support these outcomes (74, 82). Using these tools gives insight into how treatment affects behavior, not just thinking skills. They may also help parents stay involved and make it easier for children to stick with therapy, though clear evidence is still limited. While existing literature primarily emphasizes improvements in behavioral inventory scores following AT or CPAP interventions, few high-quality studies have incorporated parent-focused training or behavioral therapies into OSA management. Future research should prioritize the design and evaluation of such integrated interventions.

Overall, the future lies in bridging technological innovation with guideline-based care, ensuring early detection, personalized treatment, and long-term neurocognitive protection in children with OSA.

### 4.3 Limitations

This study has several limitations. Our bibliometric and scientometric analyses were confined to two databases (WoSCC and Scopus); consequently, relevant studies indexed in PubMed, Embase, or other sources may not have been captured. We also restricted inclusion to English-language publications, which introduces a risk of language bias and may underrepresent work from non-English-speaking regions. Bibliometric indicators such as citation counts and the h-index reflect academic visibility but do not necessarily correspond to methodological rigor or clinical relevance. For example, highly cited papers often gain recognition for technical novelty rather than direct clinical value, and recent high-quality studies may not yet be reflected in citation-based metrics. In addition, keyword and clustering analyses are shaped by authors’ terminology and database indexing practices, which can produce redundancy or obscure emerging themes.

Finally, although our review considers the potential of AI and ML in pediatric OSA, most existing models are still exploratory and have yet to undergo rigorous clinical validation. The absence of trial-level evidence linking AI-assisted diagnostics to cognitive outcomes makes it premature to draw definitive conclusions. Overall, these limitations indicate that while our study outlines major trends in this field, future work should expand database coverage, adopt standardized methodologies, and incorporate prospective validation to facilitate the translation of bibliometric insights into clinical practice.

## 5 Conclusion

Pediatric OSA is now recognized as a condition with significant impacts on cognitive development, especially in areas like attention, memory, and executive function. Sleep disruption, intermittent low oxygen levels, and coexisting conditions such as ADHD or Down syndrome contribute to ongoing neurocognitive challenges, even after standard treatments like AT or CPAP. These findings underscore the need for early detection and personalized treatment approaches.

Advances in AI and machine learning are making diagnostic tools more accessible and affordable. By analyzing physiological signals and clinical data, these techniques enable earlier risk assessment and can guide personalized treatment strategies. Although clinical application remains at an early stage, such approaches may help to address the shortcomings of conventional diagnostics and support better long-term cognitive outcomes.

Overall, research in pediatric OSA is shifting from a narrow focus on symptom relief toward mechanisms and precision-based care. The integration of technological innovation with patient-specific clinical strategies has the potential to advance pediatric sleep medicine, prioritizing durable neurocognitive protection and developmental health over short-term improvements alone.

## Data Availability

The data analyzed in this study is subject to the following licenses/restrictions: the raw datasets analyzed in this study were obtained from Web of Science and Scopus via institutional subscriptions and are not publicly available due to copyright restrictions. Processed data (e.g., extracted keywords, citation networks) are available upon reasonable request. Requests to access these datasets should be directed to Bomeng Zhao, 202500220279@sxmu.edu.cn.

## Glossary

AT: Adenotonsillectomy
AHI: Apnea–hypopnea index
AI: Artificial intelligence
AUC: Area under the curve
ANT: Attention Network Test
ADHD: Attention deficit hyperactivity disorder
BASC-2: Behavior Assessment System for Children – 2nd Edition
CHAT: Childhood Adenotonsillectomy Trial
CBCL: Child Behavior Checklist
CPAP: Continuous positive airway pressure
DN-CAS: Das–Naglieri Cognitive Assessment System
DTI: Diffusion tensor imaging
ECG: Electrocardiogram
ERP: Event-related potentials
HPA axis: Hypothalamic–pituitary–adrenal
IL-6: Interleukin-6
ML: Machine learning
MCP: Multiple-country publications
NC: Number of citations
NP: Number of publications
OSA: Obstructive sleep apnea
PATS: Pediatric Adenotonsillectomy Trial
SpO₂: Peripheral capillary oxygen saturation
PFC: Prefrontal cortex
PSG: Polysomnography
REM: Rapid eye movement sleep
ROS: Reactive oxygen species
SCP: Single-country publications
SWS: Slow-wave sleep
TNF-α: Tumor necrosis factor-*α*
WoSCC: Web of Science Core Collection
WISC: Wechsler Intelligence Scale for Children
