# Supplementary material for: Advancements in pediatric obstructive sleep apnea: cognitive implications and the role of AI in precision medicine
Source: Front Med (Lausanne). 2025 Nov 21;12:1704504. doi: 10.3389/fmed.2025.1704504 (PMC12678086; doi:10.3389/fmed.2025.1704504)
Supplement: Supplementary file 1 [file Table_1.DOCX]

**Supplementary Table S1. Bibliometric Overview of Cognition in Pediatric OSA (1983–2025)**

| **Description** | **Results** |
| --- | --- |
| MAIN INFORMATION ABOUT DATA |  |
| Timespan | 1983:2025 |
| Sources (Journals, Books, etc) | 623 |
| Documents | 1610 |
| Annual Growth Rate % | 11.96 |
| Document Average Age | 8.68 |
| Average citations per doc | 35.48 |
| References | 58525 |
| DOCUMENT CONTENTS |  |
| Keywords Plus (ID) | 7857 |
| Author's Keywords (DE) | 2892 |
| AUTHORS |  |
| Authors | 7409 |
| Authors of single-authored docs | 98 |
| AUTHORS COLLABORATION |  |
| Single-authored docs | 107 |
| Co-Authors per Doc | 5.79 |
| International co-authorships % | 10.5 |
| DOCUMENT TYPES |  |
| article | 1443 |
| review | 167 |
