# Supplementary material for: Advancements in pediatric obstructive sleep apnea: cognitive implications and the role of AI in precision medicine
Source: Front Med (Lausanne). 2025 Nov 21;12:1704504. doi: 10.3389/fmed.2025.1704504 (PMC12678086; doi:10.3389/fmed.2025.1704504)
Supplement: Supplementary file 2 [file Table_2.DOCX]

**Supplementary Table S2. Most Relevant Countries by Corresponding Author Contributions**

| Rank | **Country** | **Articles** | **Articles %** | **SCP** | **MCP** | **MCP %** |
| --- | --- | --- | --- | --- | --- | --- |
| 1 | USA | 336 | 20.9 | 290 | 46 | 13.7 |
| 2 | CHINA | 151 | 9.4 | 131 | 20 | 13.2 |
| 3 | AUSTRALIA | 64 | 4 | 56 | 8 | 12.5 |
| 4 | ITALY | 50 | 3.1 | 40 | 10 | 20 |
| 5 | KOREA | 31 | 1.9 | 29 | 2 | 6.5 |
| 6 | CANADA | 26 | 1.6 | 18 | 8 | 30.8 |
| 7 | TURKEY | 25 | 1.6 | 25 | 0 | 0 |
| 8 | UNITED KINGDOM | 25 | 1.6 | 13 | 12 | 48 |
| 9 | FRANCE | 22 | 1.4 | 15 | 7 | 31.8 |
| 10 | SPAIN | 22 | 1.4 | 9 | 13 | 59.1 |
| 11 | BRAZIL | 17 | 1.1 | 14 | 3 | 17.6 |
| 12 | JAPAN | 14 | 0.9 | 13 | 1 | 7.1 |
| 13 | NETHERLANDS | 14 | 0.9 | 9 | 5 | 35.7 |
| 14 | GERMANY | 12 | 0.7 | 6 | 6 | 50 |
| 15 | ISRAEL | 10 | 0.6 | 10 | 0 | 0 |
| 16 | NEW ZEALAND | 9 | 0.6 | 7 | 2 | 22.2 |
| 17 | BELGIUM | 7 | 0.4 | 2 | 5 | 71.4 |
| 18 | FINLAND | 6 | 0.4 | 5 | 1 | 16.7 |
| 19 | POLAND | 6 | 0.4 | 5 | 1 | 16.7 |
| 20 | SWEDEN | 6 | 0.4 | 4 | 2 | 33.3 |
| 21 | SWITZERLAND | 6 | 0.4 | 4 | 2 | 33.3 |
| 22 | CHILE | 5 | 0.3 | 2 | 3 | 60 |
| 23 | INDIA | 5 | 0.3 | 3 | 2 | 40 |
| 24 | SINGAPORE | 5 | 0.3 | 5 | 0 | 0 |
| 25 | THAILAND | 5 | 0.3 | 3 | 2 | 40 |

Abbreviation: SCP: Single Country Publications; MCP: Multiple Country Publications
